# Protective Effects of Spirulina Against Lipid Micelles and Lipopolysaccharide-Induced Intestinal Epithelium Disruption in Caco-2 Cells: In Silico Molecular Docking Analysis of Phycocyanobilin

**DOI:** 10.3390/nu16234074

**Published:** 2024-11-27

**Authors:** Fatma Arrari, Rodolfo-Matias Ortiz-Flores, Said Lhamyani, Eduardo Garcia-Fuentes, Mohamed-Amine Jabri, Hichem Sebai, Francisco-Javier Bermudez-Silva

**Affiliations:** 1 Laboratory of Functional Physiology and Valorization of Bio-Resources, Higher Institute of Biotechnology of Beja, University of Jendouba, Beja 9000, Tunisiasebaihichem@yahoo.fr (H.S.); 2 Centro de Investigacion Biomedica en Red de Diabetes y Enfermedades Metabolicas Asociadas (CIBERDEM), Instituto de Investigacion Biomedica de Malaga y Plataforma en Nanomedicina-IBIMA Plataforma BIONAND, Hospital Regional Universitario de Malaga, UGC Endocrinología y Nutricion, 29009 Malaga, Spain; 3 Centro de Investigacion Biomedica en Red de Enfermedades Hepaticas y Digestivas (CIBERehd), Instituto de Investigacion Biomedica de Malaga y Plataforma en Nanomedicina-IBIMA Plataforma BIONAND, Hospital Universitario Virgen de la Victoria, UGC de Aparato Digestivo, 29010 Malaga, Spain

**Keywords:** spirulina aqueous extract (SPAE), obesity, LPS, lipid micelles, Caco-2 cells, oxidative stress, inflammation, in silico, molecular docking, intestinal barrier

## Abstract

Damage to intestinal epithelial cells is present in obesity and other diseases because of inflammatory and oxidative processes. This damage compromises the gastrointestinal barrier, killing enterocytes, altering intestinal permeability, and eliciting abnormal immune responses that promote chronic inflammation. Recent evidence shows that spirulina is a potent natural agent with antioxidant and anti-inflammatory properties. Objectives: This study was conducted to evaluate the effect of spirulina aqueous extract (SPAE) on the alterations of the intestinal epithelium induced by lipid micelles (LMs) and/or inflammation induced by lipopolysaccharides (LPSs) in the Caco-2 cell line. Methods: In the current research, we assessed the protective actions of SPAE against cytotoxicity, oxidative stress, inflammation, and epithelial barrier perturbation by using an in vitro model, the intestinal Caco-2 cells, treated with LPSs and/or LMs. We also performed an in silico molecular docking analysis with spirulina’s bioactive compound, phycocyanobilin. Results: Our results showed that SPAE has no cytotoxic effect on Caco-2 cells. On the contrary, it improved cell viability and exhibited anti-inflammatory and antioxidant actions. SPAE also protected against endoplasmic reticulum stress and tight junction proteins, thus improving the epithelial barrier. The in silico study revealed a strong binding affinity of the phycocyanobilin compound with human SOD and NADPH oxidase and a good binding affinity towards COX-2 and iNOS. Conclusions: Taken together, these findings demonstrate the beneficial actions of SPAE on Caco-2 cells, suggesting it may be useful in preserving the epithelial intestinal barrier in human conditions involving oxidative stress and inflammation such as obesity.

## 1. Introduction

Obesity, characterized by excessive accumulation of body fat, is one of the most prevalent metabolic disorders. It is a chronic condition with a complex pathophysiology [1], typically driven by an excess of adipose tissue that results in metabolic changes and detrimental health outcomes [2]. Hyperlipidemia is a major factor contributing to obesity physiopathology, which leads to changes in the intestinal microbiota [3,4]. These microorganisms significantly influence the metabolic and immunological mechanisms of the host [5,6]. Several studies have shown that obesity reduces the levels of gram-negative and gram-positive bacteria and increases lipopolysaccharides (LPSs) in the colonic epithelium. LPSs are known to significantly contribute to both intestinal and systemic inflammatory reactions [7]. Previous research has shown that LPSs can increase the permeability of tight junctions and disrupt the intestinal barrier [8,9].

These changes contribute to compromised epithelial integrity of the colonic mucosa, resulting in increased intestinal permeability, excessive production of pro-inflammatory cytokines, reduced mucus thickness, and the development of conditions such as insulin resistance and type 2 diabetes [6,10]. Moreover, these alterations are associated with decreased gene expression of junction proteins such as zonula occludens-1 (ZO-1) and occludin (OCL-1) [5]. Among the mechanisms by which obesity directly or indirectly impacts intestinal permeability is the modification of intestinal tight junctions. Adherens junctions and intestinal tight junctions form the apical junction complex, a crucial component of the intestinal barrier [11].

In addition, obesity is linked to a state of oxidative stress, characterized by an imbalance between prooxidants and antioxidants. This imbalance leads to significant cellular damage and may result from the excessive production of reactive oxygen species (ROS) or a shortage of antioxidants [12]. ROS damage tight junction proteins by oxidizing them, which alters their structure and function, resulting in increased epithelial permeability, commonly referred to as “leaky gut” [13,14]. This disruption weakens the tight junctions, compromising the epithelial barrier and allowing harmful substances to pass through.

Hyperlipidemia is closely tied to chronic adipose tissue damage, caused by fatty acid buildup and the transformation of monocytes into macrophages, which sustain local injury [15]. M1 macrophages release cytokines like TNF-α, IL-1, and IL-6, driving T lymphocyte recruitment [16]. Studies show that being overweight is strongly linked to the abnormal expression of pro-inflammatory cytokines such as leptin, TNF-α, and IL-6, along with other markers like serum amyloid, CRP, and fibrinogen [17]. These cytokines directly affect the structure and function of tight junctions. They disrupt the interactions between tight junction proteins such as occludin, claudin, and zonula occludens, leading to altered permeability [18,19].

Traditional therapeutic remedies were predominant and practiced for centuries until the nineteenth century saw the rise of synthetic medications [20]. In recent years, attention has increasingly focused on the potential of various microalgae, which are prized for their strong pharmacological properties, few side effects, and cost-effectiveness [21]. Spirulina is a spiral-shaped blue-green microalga with a filamentous structure [22,23]. It is renowned for its rich composition of phytochemicals, proteins, antioxidants, vitamins, fiber, amino acids, and minerals. C-phycocyanin [24], which contains phycocyanobilin (PCB), is the dominant pigment in spirulina, making up a significant portion of its composition. It contributes to its antioxidant capacity, helping to neutralize free radicals and reduce oxidative stress [25]. Additionally, PCB has been associated with various health benefits, including anti-inflammatory, immunomodulatory, and neuroprotective effects [26]. Spirulina’s high content of phenolic compounds, such as chlorogenic acid, syringic acid, and sinapic acid, contributes to its regulatory effects on lipid and oxidative stress metabolism [27].

Spirulina is increasingly recognized for its potential therapeutic benefits against various diseases, owing to its potent anti-inflammatory, antitumor, antioxidant, antiviral, and antibacterial properties [28,29]. Recent studies suggest that microalgae, including Spirulina, could play a crucial role in managing hyperlipidemia. They have been found to influence appetite regulation, insulin sensitivity, oxidative balance, nutrient absorption, and infection control [30,31]. Clinical trials have demonstrated Spirulina’s ability to reduce total cholesterol, triglyceride, and LDL cholesterol levels in humans [32]. Hence, this study was conducted to evaluate the effect of spirulina aqueous extract (SPAE) on the alterations of the intestinal epithelium induced by lipid micelles (LMs) and/or inflammation induced by LPSs in a well-established model of the human enterocyte, the Caco-2 cell line. Furthermore, we performed docking studies to provide crucial insights into the potential binding modes of the PCB compound from spirulina within the active sites of the nicotinamide adenine dinucleotide phosphate (NADPH) oxidase, superoxide dismutase (SOD), cyclooxygenase-2 (COX-2), and inducible nitric oxide synthase (iNOS) enzymes.

## 2. Materials and Methods

### 2.1. Chemicals

Bovine catalase, Random Primers, butylated hydroxytoluene, DMEM (Dulbecco’s Modified Eagle’s Medium), Trizol reagent, reverse transcriptase, 2-Thio-barbituric acid, pyrogallol, PBS, trypsine-EDTA, FD-4, fœtal bovine serum, penicillin, streptomycin, chloroform, palmitic acid, taurocholic acid, cholesterol, alcohol isopropyl, methanol, chlorhydric acid (HCl), EDTA, and hydrogen peroxide were purchased from Sigma-Aldrich Co., Munich, Germany.

### 2.2. Spirulina Aqueous Extract Preparation (SPAE)

The spirulina flakes were sourced from the Tunisian company Eden Life (Kettana, Tunisia). These flakes were then ground into a fine powder using an electric blender (Oster, Newell Brands, Atlanta, GA, USA). The resulting powder was placed into bottles and stored in a dry, dark environment. Five grams of spirulina powder was mixed with 50 mL of distilled water and incubated at room temperature for 24 h with continuous magnetic stirring. The mixture was then centrifuged at 10,000× *g* for 10 min and filtered [33]. The resulting supernatant was lyophilized using a refrigerated Vapor Trap RVT450 (Thermo Fisher Scientific, Waltham, MA, USA). The lyophilized product (SPAE) was then divided into aliquots and stored in the dark until needed.

### 2.3. Cell Culture and Treatments

The human colonic cancer cell line Caco-2 was available at the Endocrinology and Nutrition Unit at the Regional Hospital of Malaga (IBIMA, Spain). Owing to their ability to differentiate into a monolayer with typical enterocyte properties, Caco-2 cells were used as an in vitro model of the intestinal barrier. The Caco-2/TC7 cell line is a clonal population of Caco-2 cells derived from human colonic carcinoma that exhibit significant morphological, characteristic, and functional characteristics typical of enterocytes [34]. Throughout the treatment, the cells were seeded and maintained in DMEM enriched with 10% fœtal bovine serum, 4 mM L-glutamine, 1 mM pyruvic acid, 4.5 mg/mL D-glucose, 100 U/mL penicillin G, and 100 µg/mL streptomycin sulfate at a temperature of 37 °C in a humid atmosphere with 5% CO_2_. The cells were cultured on 24-well Transwell^®^ filters (Thermo Fisher Scientific, Waltham, MA, USA) at a density of 0.2 × 10^6^ cells/well for 3 weeks to obtain fully differentiated enterocyte-like cells (the medium was replaced every 3 days). After 21 days and 100% confluence, the Caco-2 cells were incubated with 375 µL of LM added to the apical medium for 4 consecutive days. During the addition of the micelles, a DMEM volume was removed and the LM in the apical compartment was replaced. Subsequently, 250 µL of LPS (1 µg/mL) was added to the upper compartment of the plate, which was incubated for 3 h at 37 °C. After 3 h, spirulina aqueous extract (SPAE 500 µg/mL) was added for 24 h while the same volume of PBS was added to the untreated groups. We used aqueous extracts created with milder methods compared to organic solvents, which help preserve sensitive nutrients, vitamins, and antioxidants. These extracts dissolve effectively in water, enhancing the bioavailability of spirulina’s bioactive compounds for improved absorption in the gastrointestinal tract. Previous studies reported the absence of cytotoxic effects up to 500 µg/mL. The concentration of 500 μg/mL was chosen based on prior in vitro studies, where similar concentrations of spirulina extracts have demonstrated notable anti-inflammatory and antioxidant effects, as reported in a recent study by Sowmya et al. [35].

At the end of the experiment, the basal and apical media were collected to measure different biomarkers, and the cells were then washed with PBS and harvested by trypsinization for the analysis of various biochemical parameters [36]. The eight experimental groups are outlined below:

1. Control: Caco-2 cells in DMEM without treatment.
2. LM: Caco-2 cells incubated with LM (4 days) without treatment.
3. LM + SPAE: Caco-2 cells incubated with LM (4 days) and treated with SPAE (500 µg/mL) for 24 h.
4. SPAE: Caco-2 cells in DMEM treated with SPAE (500 µg/mL) for 24 h.
5. LPS: Caco-2 cells incubated with LPS (1 µg/mL) for 3 h.
6. LPS + SPAE: Caco-2 cells incubated with LPS (1 µg/mL) for 3 h and treated with SPAE (500 µg/mL) for 24 h.
7. LPS + LM: Caco-2 cells incubated with LM (4 days) and LPS (1 µg/mL) for 3 h.
8. LPS + LM + SPAE: Caco-2 cells incubated with LM (4 days) and LPS (1 µg/mL) for 3 h and treated with SPAE (500 µg/mL) for 24 h.

### 2.4. Preparation of Lipid Micelles

Palmitic acid (Sigma-Aldrich, St. Louis, MO, USA) was provided in the form of complex micelles to mimic its physiological form as found in the intestinal lumen in vivo. Other complex micelles (2 mmol sodium taurocholate, 0.6 mmol oleic acid or 0.6 mmol palmitic acid, 0.2 mmol lysophosphatidylcholine, 0.05 mmol cholesterol, and 0.2 mmol monoacylglycerol) were prepared and added to the upper compartment for specified durations. The micelles were removed and replaced with fresh apical medium during the epithelial integrity analysis [37,38].

### 2.5. Intestinal Permeability Measurements in Caco-2 Cells

At the end of the treatment, the paracellular permeability of the Caco-2 monolayer was evaluated by measuring the unidirectional flux of fluorescein isothiocyanate-labeled dextran-4 (FD4) from the apical to the basolateral compartment of the Transwell. Twenty microliters of FD4 (5 mg/mL stock solution) was added to the apical medium. Then, 480 µL of PBS was added to achieve a final concentration of 1 mg/mL. After 3 h, aliquots of 100 µL from the basolateral compartment were collected every 30 min for 3 h and replaced with an equal volume of PBS in the apical compartment [39]. Aliquots from each 30 min interval were stored on ice. For the final aliquot, samples were transferred to a black 96-well plate. The concentration of FD4 was determined using a fluorescence plate reader (BioTek Instruments, Winooski, VT, USA) with an excitation wavelength of 480/492 nm and an emission wavelength of 520/525 nm against a 100% PBS blank. The apparent permeability (Papp) for each assay was calculated using the following equation:

$$Papp\left(\mathrm{cm}/s\right)=\left(\frac{dQ}{dt}\right)*\left(\frac{1}{A}\right)*C0$$

- Papp = apparent permeability (cm/s)
- dQ/dt = permeability rate (amount permeated per minute over the 240 min duration)
- A = area of monolayer diffusion (cm^2^)
- C0 = initial concentration of the studied compounds.

The flux term dQ/dt was calculated using linear regression of the amounts in the receiving chamber over time.

### 2.6. Viability/CytotoxicityWST-1assay

The WST-1 assay (Takara Bio USA, Mountain View, CA, USA) is a colorimetric method used to assess the viability of Caco-2 cells treated with LM, LPS, and SPAE (500 µg/mL). Briefly, 2 × 10^7^ cells were cultured in a 96-well plate with 100 µL of DMEM and incubated for 24 h. The following day, the cells were treated with lipid micelles for 4 days. Subsequently, LPS was added, and the cells were incubated for 3 h at 37 °C. After 3 h, SPAE (500 µg/mL) was added and the mixture was incubated for 24 h. At the end of the test, 10 µL of WST-1 reagent was added to each well and incubated for 4 h at 37 °C. The plates were then read at 450 nm using a spectrophotometric plate reader (BioTek Instruments, Inc., Winooski, VT, USA) [40]. The percentage (%) of cell viability was calculated via the following equation:

$$Cell\;viability\left(\%\right)=\frac{OD\;treated}{OD\;control}*100$$

where OD treated is the absorbance of the cells treated with different treatments and OD control is the absorbance of control cells.

### 2.7. RNA Extraction

After treatment, the cells were washed with PBS and 1 mL of Trizol was added to each well. Total RNA isolation was performed using the mirVana™ mRNA Kit (Ambion Life Technologies, Carlsbad, CA, USA) according to the manufacturer’s protocol. The concentration and purity of the extracted RNA were determined using a NanoDrop1000 spectrophotometer (Thermo Fischer Scientific, Inc., Waltham, MA, USA). The samples had A260/A280 absorbance ratios ranging from 1.8 to 2.1, indicating highly pure RNA. The samples were then stored at −80 °C until used for complementary DNA (cDNA) synthesis.

### 2.8. cDNA Synthesis

cDNA was synthesized using the TaqMan^®^ MicroRNA Reverse Transcription Kit (Promega Biotech Ibérica S.L., Madrid, Spain), with specific primers and probes for each mRNA (TaqMan^®^ MicroRNA Assay, Applied Biosystems, Foster City, CA, USA). The reverse transcription program consisted of 30 min at 16 °C, 30 min at 42 °C, and 5 min at 85 °C (to deactivate DNA polymerase). The samples were then stored at −80 °C until analysis by RT-qPCR.

### 2.9. Gene Expression Quantification by Real-Time qPCR (RT-qPCR)

The expression levels of the mRNAs were evaluated by RT-qPCR using the Applied Biosystems 7500 Fast Real-Time PCR System (Applied Biosystems, CA, USA) and SYBR Green master mix (Life Technologies, CA, USA) following the manufacturer’s protocol. Each sample was evaluated in triplicate, and the relative quantification of mRNA levels was performed using the comparative threshold cycle (Ct) method according to the manufacturer’s guidelines. The primer sequences used in this study are presented in Table 1. The experimental protocol included the following programs: one cycle at 95 °C for 5 min, followed by 40 cycles at 95 °C for 15 s and 60 °C for 30 s. The melting curves of all the PCR products were evaluated to confirm a single amplification product with the expected melting temperature. A comparative method (2^−ΔCT^) was used to analyze the relative expression of the genes of interest. GAPDH was used as a reference gene, and all the data were normalized.

### 2.10. Biochemical Analysis of Oxidative Stress Markers

#### 2.10.1. Protein Assay

The protein content was measured according to [41]. This method is based on the ability of the protein-copper complex to reduce Folin’s reagent, resulting in a blue color at 650 nm. After constructing the calibration curve (optical density versus protein quantity in μg), the protein concentration of the assays was determined by plotting the optical density against the calibration curve using bovine serum albumin as a standard.

#### 2.10.2. Oxidative Stress Assessment

Malondialdehyde (MDA) levels were measured following the method outlined by Draper and Hadley [42], which involves the reaction of MDA with thiobarbituric acid. Thiol group (-SH) estimation was conducted in accordance with Ellman’s method [43], whereas reduced glutathione (GSH) levels were evaluated via the Sedlak and Lindsay method [44]. SOD activity was assessed following the procedure described by Misra and Fridovich, utilizing the epinephrine/adrenochrome system [45]. The protocol outlined by Flohé and Günzler was employed to examine Glutathione peroxidase (GPx) activity [46], and catalase (CAT) activity was determined using the method described by Aebi [47].

#### 2.10.3. Reactive Oxygen Species Measurement

Hydrogen peroxide (H_2_O_2_) was determined according to the method of Dingeon et al. (1975) [48]. Briefly, a reaction medium containing H_2_O_2_, phenol, and peroxidase transforms 4-Aminoantipyrine into Quinoneimine, which is pink in color and is observed at 505 nm. Calculations were performed following a calibration curve plotted from a series of standard hydrogen peroxide mixtures.

The hydroxyl radical level was determined according to the method of Payá et al. (1987) [49]. Briefly, incubation of the cells supernatant with 2-deoxyribose for 60 min at 37 °C causes oxidation by the hydroxyl radical. The reaction was halted by adding 2.8% Trichloroacetic acid and 1% thiobarbituric acid and boiling for 20 min at 100 °C. Changes in absorbance were noted at 532 nm against a blank composed of desoxyribose and buffer.

The superoxide anion level was measured using the Marklund and Marklund method (1974) [50] with slight modifications. Briefly, the homogenates were incubated in Tris-HCl buffer, and then pyrogallol was added and the mixture was incubated at 25 °C for 4 min. The reaction was stopped by adding HCl, and the absorbance was read at 420 nm against the blank.

### 2.11. Quantification of Cytokines by ELISA

The concentrations of pro-inflammatory cytokines TNFα, IL-6, and IL-1β were assessed using ELISA kits from Cambridge Bioscience (Cambridge Bioscience Limited, Cambridge, UK) following the manufacturer’s guidelines. For the in vitro treatment of Caco-2/TC7 cells, 0.2 mL of basal medium was used for the ELISA procedure.

### 2.12. Molecular Docking Study

The molecular docking procedure was used to study PCB and its interactions with targeted proteins, focusing on the types of intermolecular interactions and their binding mode energies. PCB is a chromophore present in blue-green algae and cyanobacteria such as Spirulina and has been identified as a powerful inhibitor of numerous enzyme complexes [51]. Initially, the 3D crystal structures of NADPH oxidase (PDB ID: 2CDU), SOD (PDB ID: 4MCM), COX-2 (PDB ID: 3LN1), and iNOS (PDB ID: 1NSI) were obtained from the RCSB Protein Data Bank (PDB ID; https://www.rcsb.org/; accessed 12 July 2024), and the 3D structures of our molecule were obtained from PubChem (https://pubchem.ncbi.nlm.nih.gov/; accessed 12 July 2024). The NADPH oxidase and SOD structures were specifically selected to investigate the antioxidant activity, whereas COX-2 and iNOS were chosen to evaluate the anti-inflammatory properties of PCB compounds. The proteins were processed primarily using AutoDock 4.2 software. This process involves removing all co-crystallized ligands from proteins, adding Kollman charges, and eliminating any water molecules [52]. The docking system against NADPH oxidase, SOD, COX-2, and iNOS used a grid box centered on the target protein with dimensions of 60 × 60 × 60 and a spacing of 0.375 Å. The grid center coordinates (x, y, z) were set as follows: for NADPH oxidase, 12.41, 2.25, 15.15; for SOD, 8.59, 126.10, 15.48; for COX-2, 28.06, −20.88, −13.27; and for iNOS, 9.32, 65.86, 23.01. The two- and three-dimensional interactions of the strongest ligand–receptor complexes were visualized using Discovery Studio 2021 software [53].

### 2.13. Statistical Analysis

Statistical analysis was conducted using Statistica 13.0 data analysis software (TIBCO Software Inc., Palo Alto, CA, USA). Before analysis, all the results were assessed for normality and homoscedasticity to ensure the validity of the subsequent tests. To evaluate the impact of LM and LPS on cellular viability, permeability with FD-4, ER stress markers, intercellular tight junction proteins, and iNOS, a one-way ANOVA was performed. This was followed by a post hoc least significant difference (LSD) test to pinpoint specific differences between groups. Antioxidant parameters and lipid peroxidation levels in each treatment group were assessed using the non-parametric Kruskal–Wallis test, followed by Mann–Whitney post hoc analysis. Statistical significance was determined at thresholds of *p* < 0.05. These levels of significance were represented as * for comparisons with the control; ¥ for comparisons with the LM; # for comparisons with the LPS; and £ for comparisons with the LM + LPS.

## 3. Results

### 3.1. Effects of SPAE, LPS, and/or Lipid Micelles on Cell Viability

**Figure 1:**
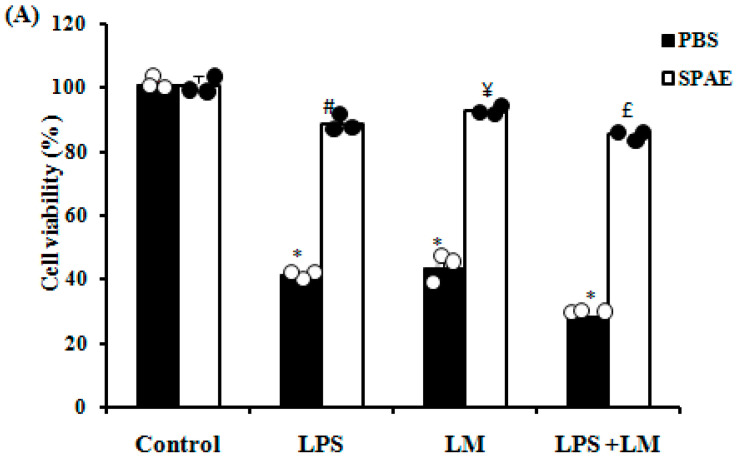

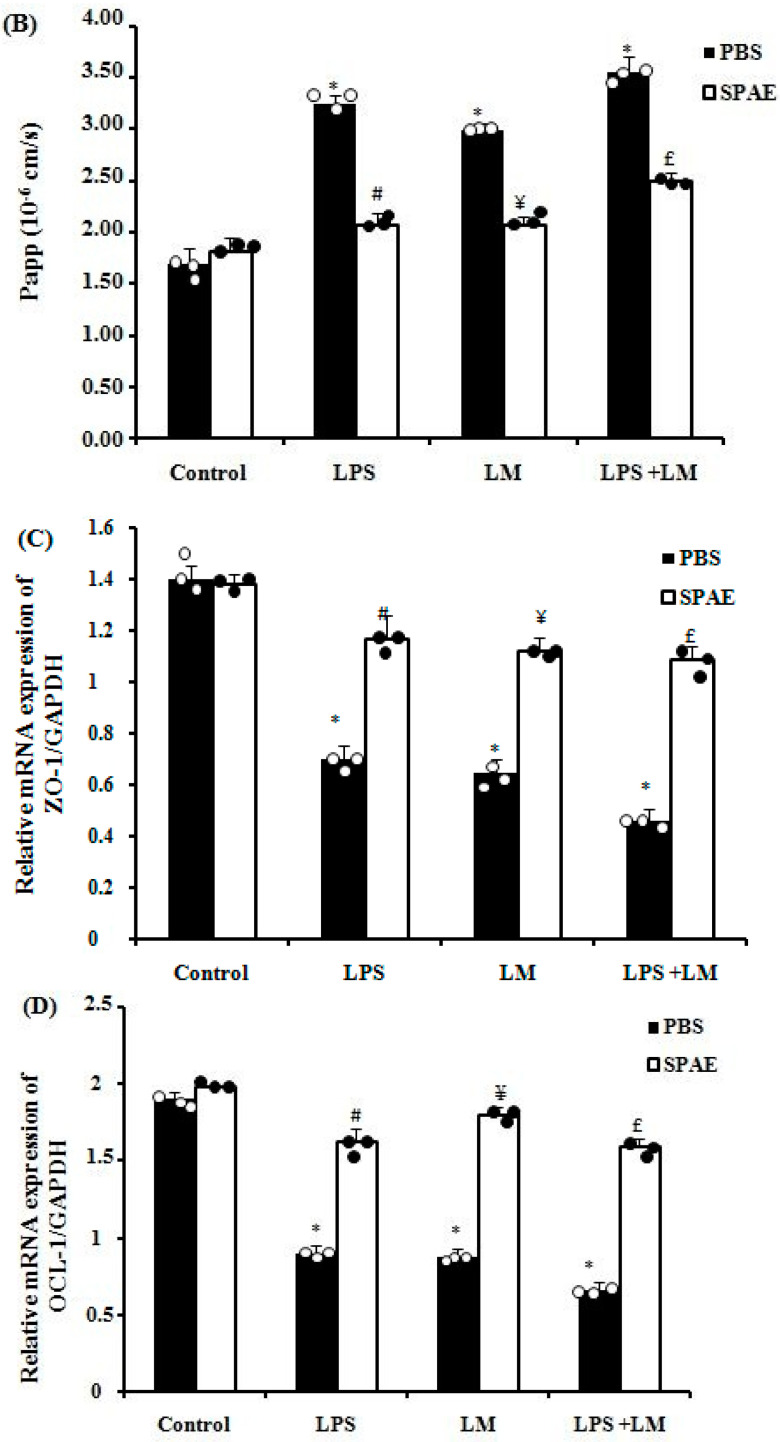
Effects of SPAE (500 μg/mL) on the viability (**A**), the epithelial barrier permeability (**B**), and gene expression of tight junction proteins ZO-1 (**C**) and OCL-1 (**D**) of intestinal Caco-2 cells challenged with LPS (1 μg/mL) and/or LM. The WST-1 assay and the FD-4 assay were performed after 24 h of incubation with SPAE. Cells were incubated with LM for 4 days and/or LPS for 3 h in the presence or absence of SPAE. Results are expressed as mean ± SEM (n = 3). *: *p* < 0.05 vs. Control; #: *p* < 0.05 vs. LPS; ¥: *p* < 0.05 vs. LM; and £: *p* < 0.05 vs. LPS + LM. One-way ANOVA followed by a post hoc LSD test.

The results showed that LPS and/or LM significantly decreased the viability of Caco-2 cells (Figure 1A) compared to the control group. SPAE administration protected against the deleterious effects of LPS and/or LM on cell viability. Spirulina alone, at a concentration of 500 μg/mL, showed no significant cytotoxic effects after 24 h.

### 3.2. Effects of SPAE, LPS, and/or LM on Epithelial Barrier Permeability

To demonstrate the impact of LM and LPS on the epithelial cell compartment, we evaluated the effects of SPAE, LPS, and/or LM on the paracellular permeability of a human Caco-2 cell monolayer using FD4, which showed a significant increase in the fluorescence intensity of FD4 applied to the apical compartment of the cell monolayer treated with LPS and/or LM compared with that of the control. Interestingly, SPAE protects against fluorescence intensification induced by endotoxins and/or saturated fatty acids (Figure 1B).

### 3.3. Effects of SPAE, LPS, and/or Lipid Micelles on Intercellular Tight Junction Proteins

We also examined the expression levels of tight junction protein complexes (ZO-1 and OCL-1) by RT-qPCR to determine the mechanisms underlying the protective effect of spirulina aqueous extract on epithelial paracellular barrier integrity. As shown in Figure 1 mRNA expression levels of ZO-1 (Figure 1C) and OCL-1 (Figure 1D) significantly decreased after cell toxicity with LPS, LM, or their combination compared with the control group. In contrast, the administration of SPAE significantly corrected the overexpression of tight junction protein levels.

### 3.4. LPS and/or LM-Induced Increase in Endoplasmic Reticulum (ER) Stress Markers Expression in Caco-2 Cells Is Prevented by SPAE

**Figure 2:**
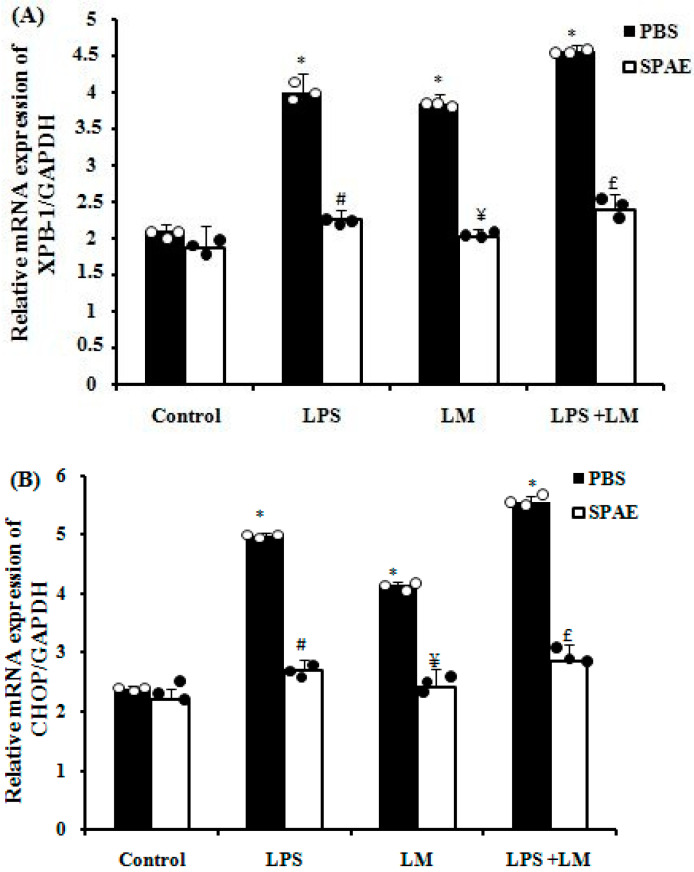
Effects of SPAE (500 μg/mL) on the gene expression of the ER stress markers XBP-1 (**A**) and CHOP (**B**) after LPS (1 μg/mL) and/or LM in cultured Caco-2 intestinal cells. Cells were incubated with LM for 4 days and/or LPS for 3 h in the presence or absence of SPAE. Results are expressed as mean ± SEM (n = 3). *: *p* < 0.05 vs. Control; #: *p* < 0.05 vs. LPS; ¥: *p* < 0.05 vs. LM; and £: *p* < 0.05 vs. LPS + LM. One-way ANOVA followed by a post hoc LSD test.

After incubation with lipid micelles for 4 consecutive days and/or LPS for 3 h, RT-PCR analysis revealed significant transcriptional overexpression of ER stress markers such as XBP-1 and CHOP (Figure 2A,B). In contrast, co-incubation with SPAE (500 μg/mL) reverted this overexpression (Figure 2).

### 3.5. Effects of SPAE, LPS, and/or LM on Inducible Nitric Oxide Synthase (iNOS) and Pro-Inflammatory Cytokine Expression

**Figure 3:**
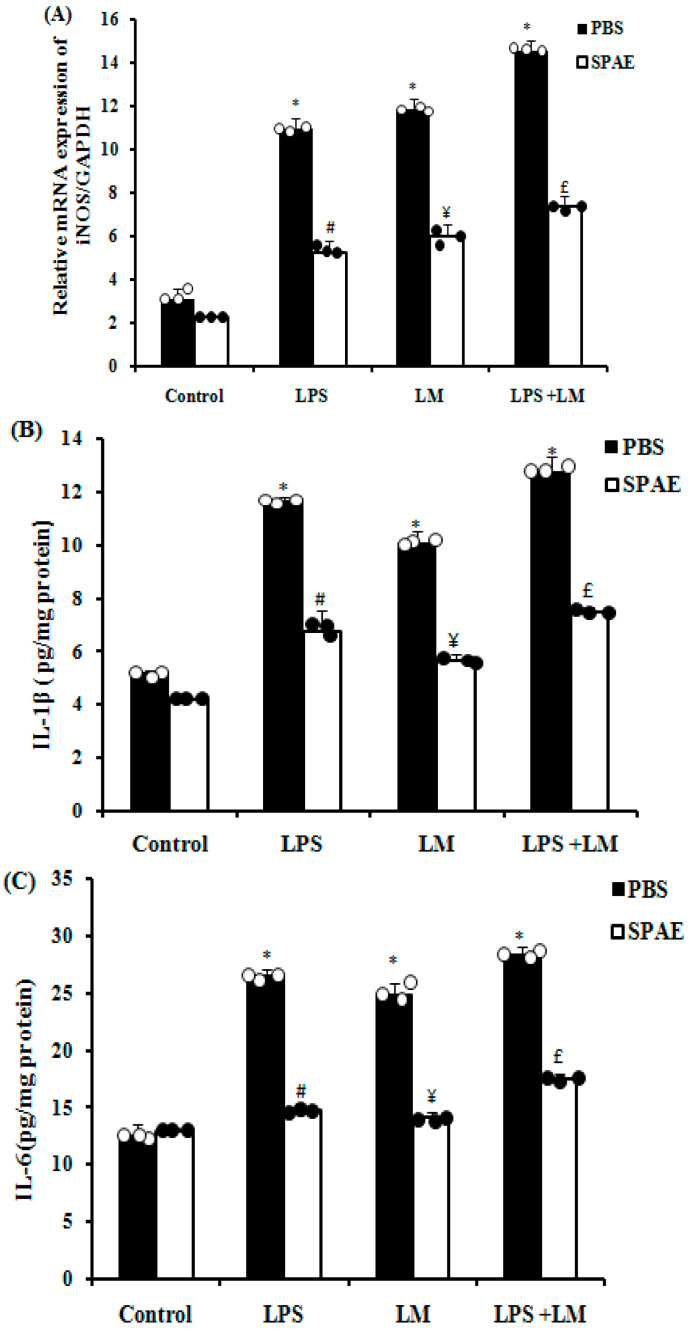

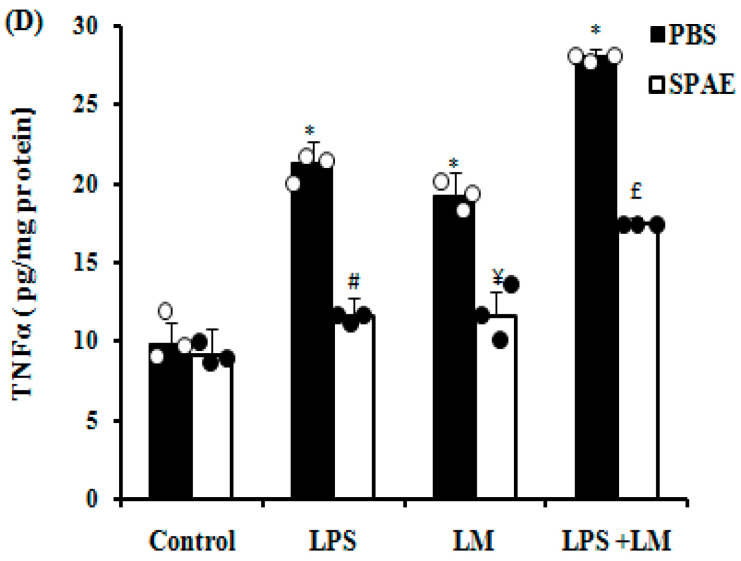
Effects of SPAE (500 μg/mL) on iNOS enzyme gene expression (**A**) and the production of the pro-inflammatory cytokines IL-1β (**B**), IL-6 (**C**), and TNFα (**D**) in cultured Caco-2 cells challenged with LPS (1 μg/mL) and/or LM. Cells were incubated with LM for 4 days and/or LPS for 3 h in the presence or absence of SPAE. Results are expressed as mean ± SEM (n = 3). *: *p* < 0.05 vs. Control; #: *p* < 0.05 vs. LPS; ¥: *p* < 0.05 vs. LM; and £: *p* < 0.05 vs. LPS + LM. One-way ANOVA followed by a post hoc LSD test.

Our results revealed a significant increase in iNOS mRNA expression following LPS or LM incubation. The effect was more marked with the combination of the two insults. The administration of SPAE significantly down-regulated this overexpression (Figure 3A). LPS and LM caused a significant increase in the levels of pro-inflammatory cytokines such as IL-1β (Figure 3B), IL-6 (Figure 3C), and TNFα (Figure 3D) compared with the control group, being more marked in cells treated with the combination of LPS and LM. More importantly, treatment with SPAE effectively protected against endotoxin- and saturated fatty acid-induced overexpression of pro-inflammatory cytokines.

### 3.6. Effects of SPAE, LPS, and/or LM on Oxidative Parameters

#### 3.6.1. Enzymatic Antioxidants (SOD, CAT, and GPx)

**Figure 4:**
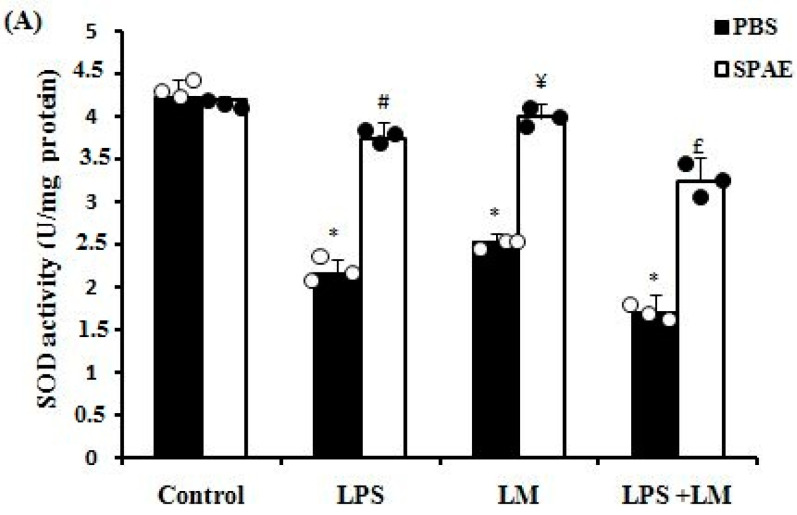

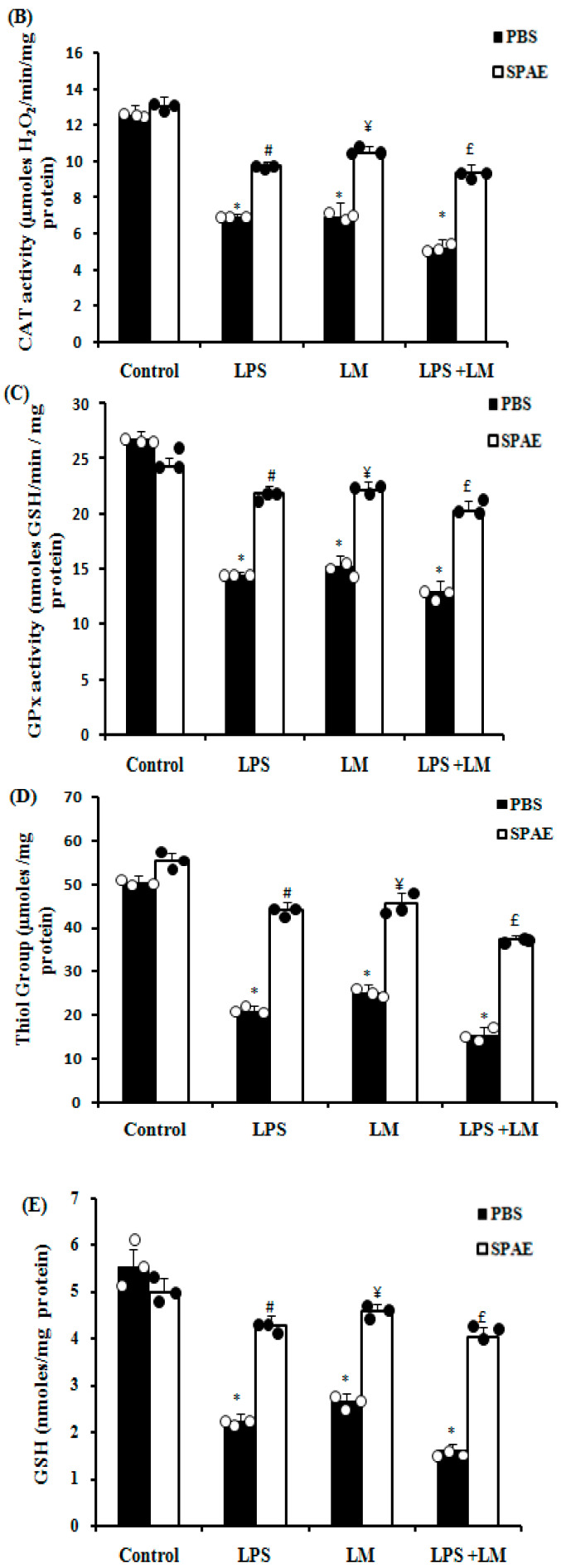
Effects of SPEA (500 μg/mL) on the activity of antioxidant enzymes such as SOD (**A**), CAT (**B**), and GPx (**C**) and the levels of non-enzymatic antioxidants such as thiol groups (**D**) and reduced glutathione (**E**) in cultured Caco-2 cells after LPS (1 μg/mL) and/or LM. Cells were incubated with LM for 4 days and/or LPS for 3 h in the presence or absence of SPAE. Results are expressed as mean ± SEM (n = 3). *: *p* < 0.05 vs. Control; #: *p* < 0.05 vs. LPS; ¥: *p* < 0.05 vs. LM; and £: *p* < 0.05 vs. LPS + LM. Non-parametric Kruskal–Wallis test, followed by Mann–Whitney post hoc analysis.

In the same context, we saw that the LPS, LM, and LPS + LM groups showed a significant depletion in the activity of antioxidant enzymes such as SOD (Figure 4A), CAT (Figure 4B), and GPx (Figure 4C) compared with the control group. Treatment with SPAE significantly restored the activity of these enzymes.

#### 3.6.2. Non-Enzymatic Antioxidants (G-SH and GSH)

We also found that LPS and/or LM induced a significant decrease in the levels of thiol groups (Figure 4D) and GSH (Figure 4E) compared with the control group. The administration of SPAE significantly protected against this disruption of non-enzymatic antioxidants.

#### 3.6.3. Lipid Peroxidation

**Figure 5:**
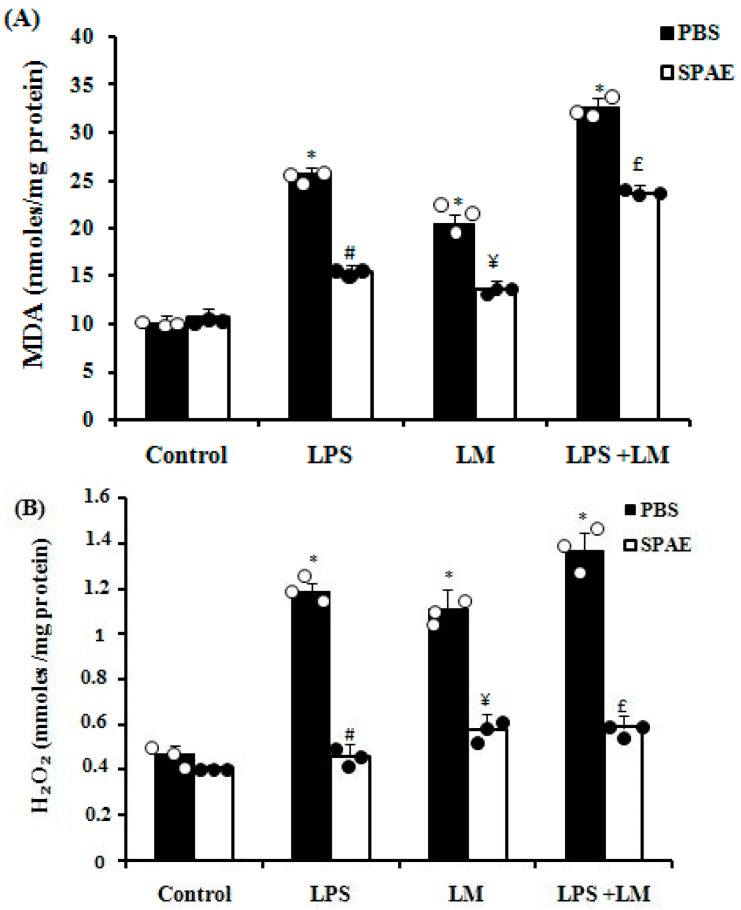

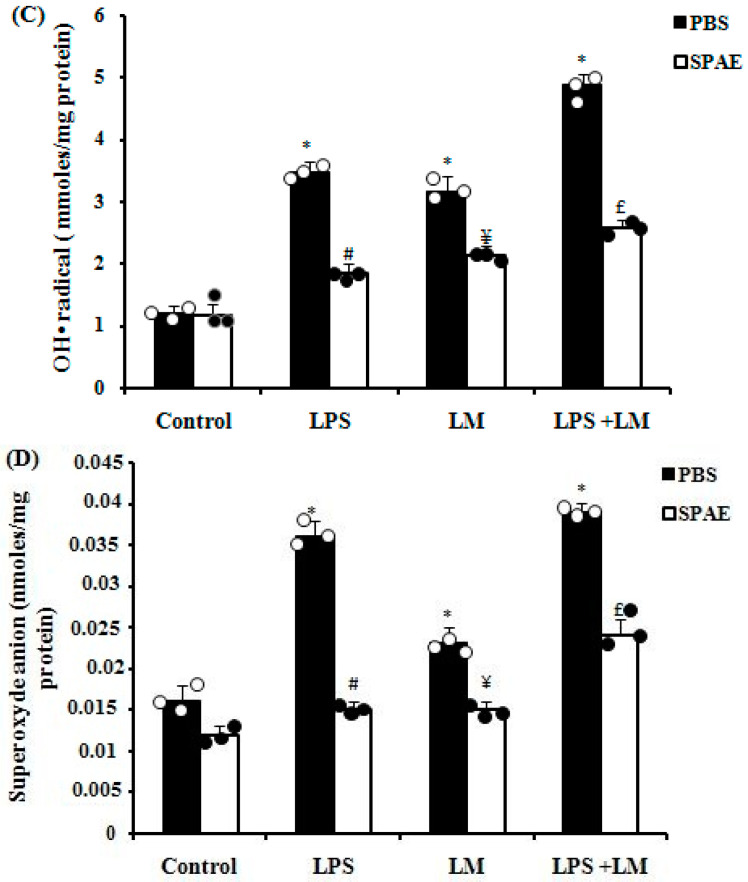
Effects of SPAE (500 μg/mL) on the membrane lipoperoxidation (MDA levels) (**A**) and the levels of reactive oxygen species such as hydrogen peroxide (**B**), hydroxyl radical (**C**), and superoxide anion (**D**) in cultured Caco-2 cells after LPS (1 μg/mL) and/or LM. Cells were incubated with LM for 4 days and/or LPS for 3 h in the presence or absence of SPAE. Results are expressed as mean ± SEM (n = 3). *: *p* < 0.05 vs. Control; #: *p* < 0.05 vs. LPS; ¥: *p* < 0.05 vs. LM; and £: *p* < 0.05 vs. LPS + LM. Non-parametric Kruskal–Wallis test, followed by Mann–Whitney post hoc analysis.

LM, LPS, and their combination induced lipid peroxidation at the cellular level, shown by a significant increase in MDA levels compared with the control group (Figure 5A). The administration of SPAE significantly protected against the lipoperoxidation of cell membranes (Figure 5A). 

#### 3.6.4. Effects of SPAE, LPS, and/or LM on Reactive Oxygen Species (ROS) Production in Cultured Caco-2 Cells

Induction of inflammation by LPS and/or LM in cultured Caco-2 cells elicited an overload of reactive oxygen species in the cell monolayer, as assessed by increased levels of hydrogen peroxide (Figure 5B), hydroxyl radicals (Figure 5C), and superoxide anions (Figure 5D). In contrast, treatment with SPAE showed potent ROS-scavenging activity, with a significant decrease in ROS overexpression at the cellular level (Figure 5B–D).

### 3.7. In Silico Docking Study

**Figure 6:**
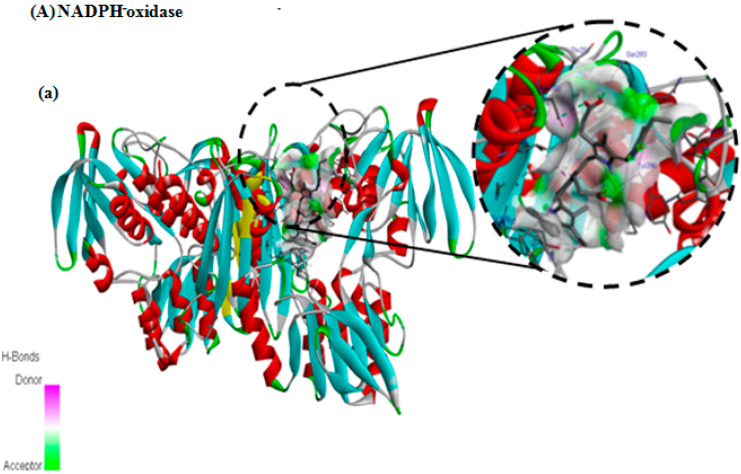

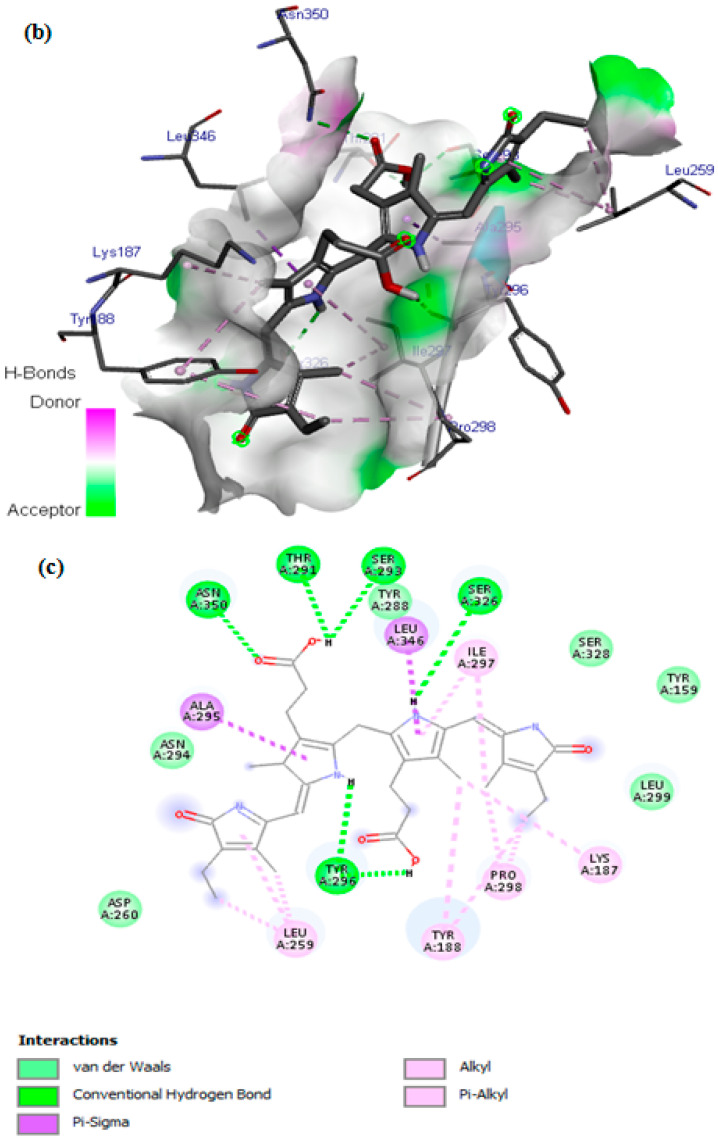

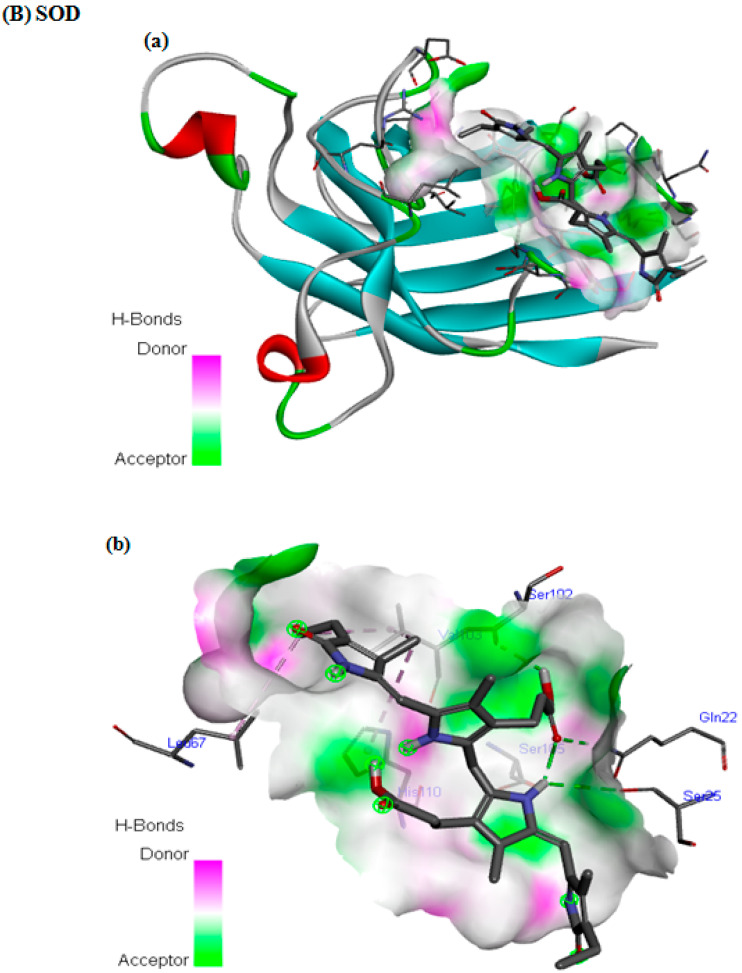

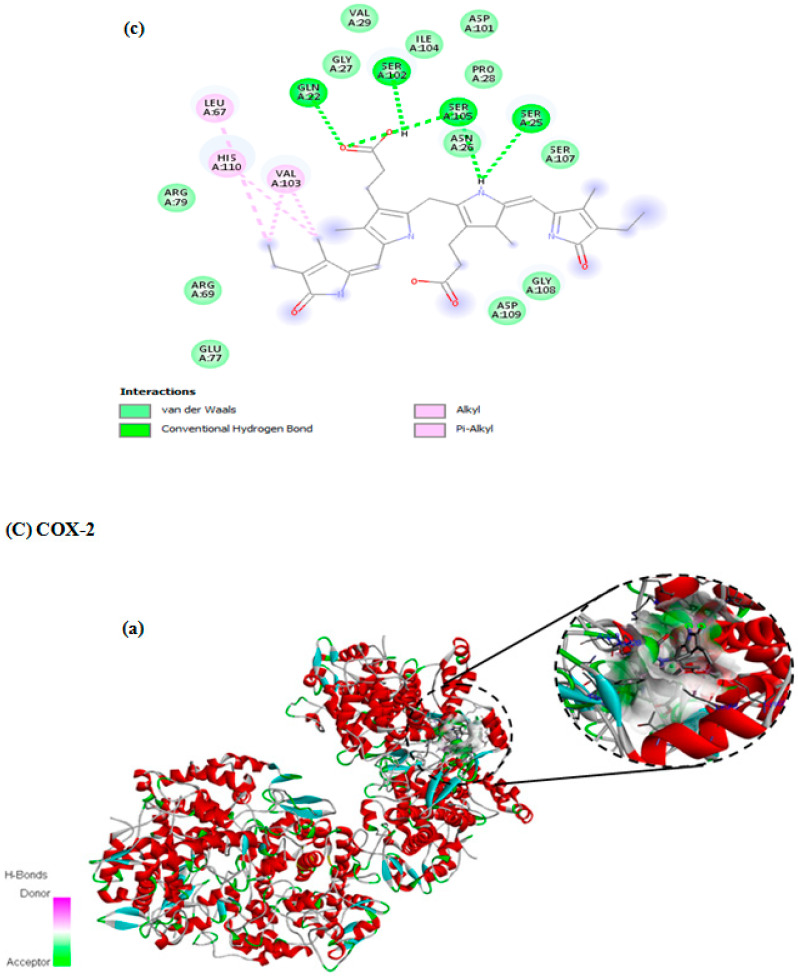

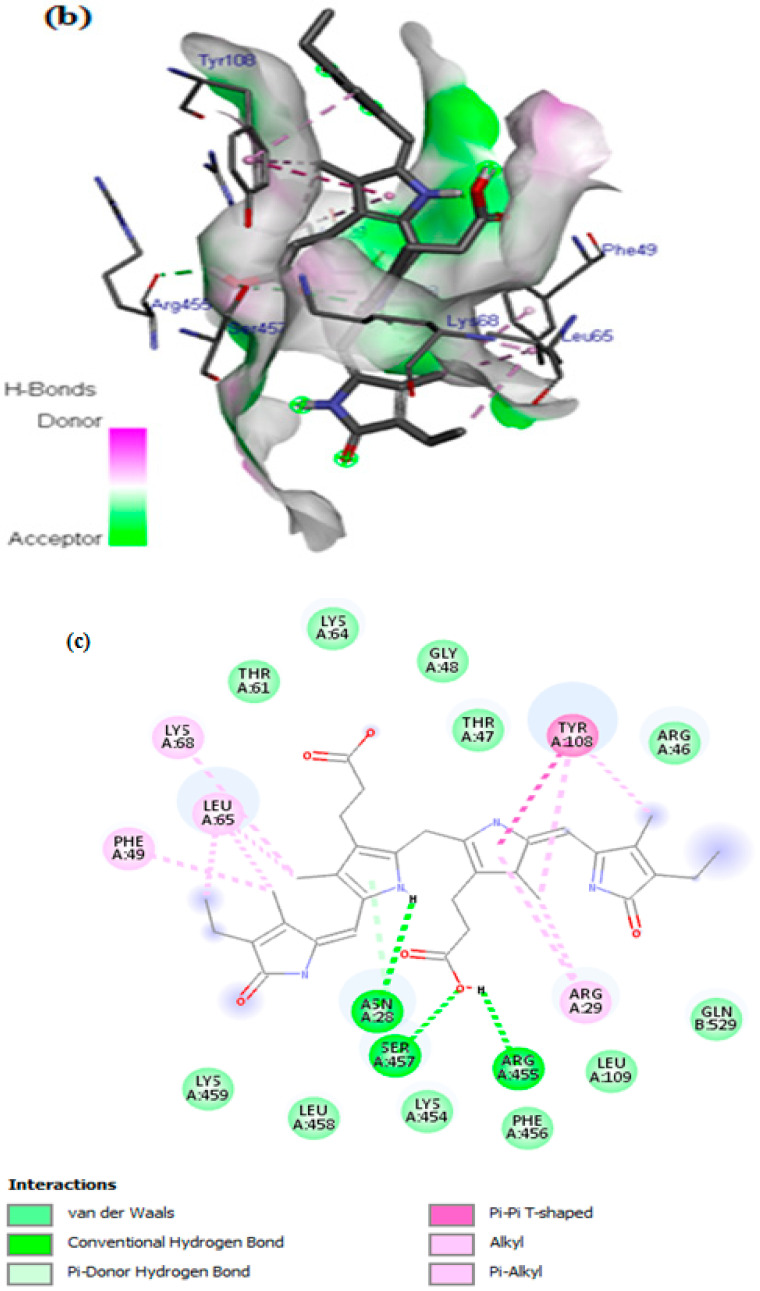

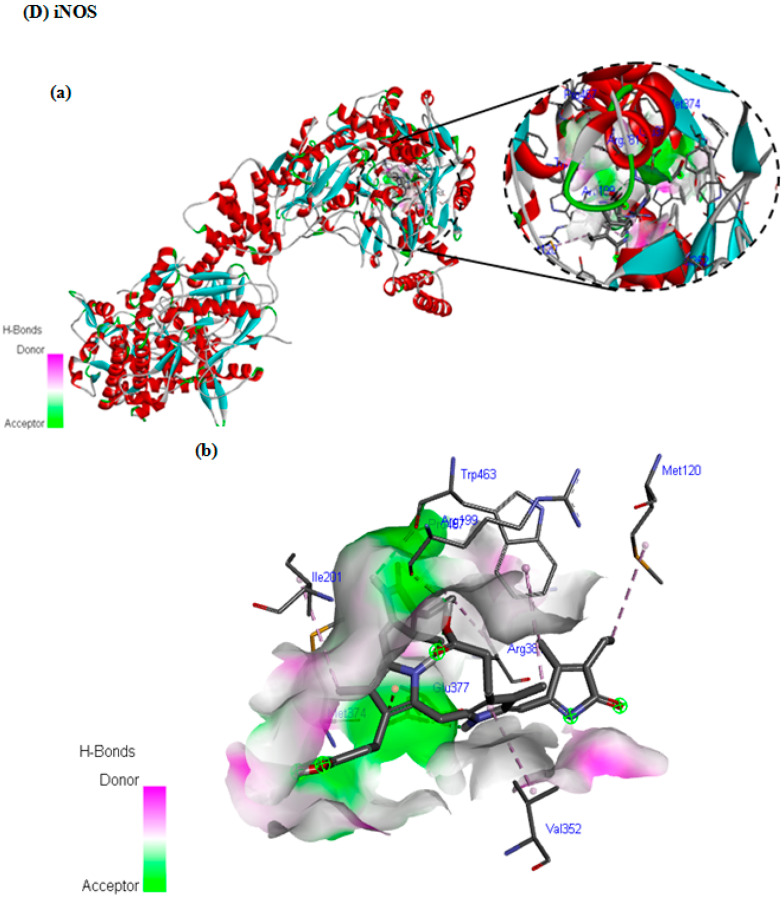

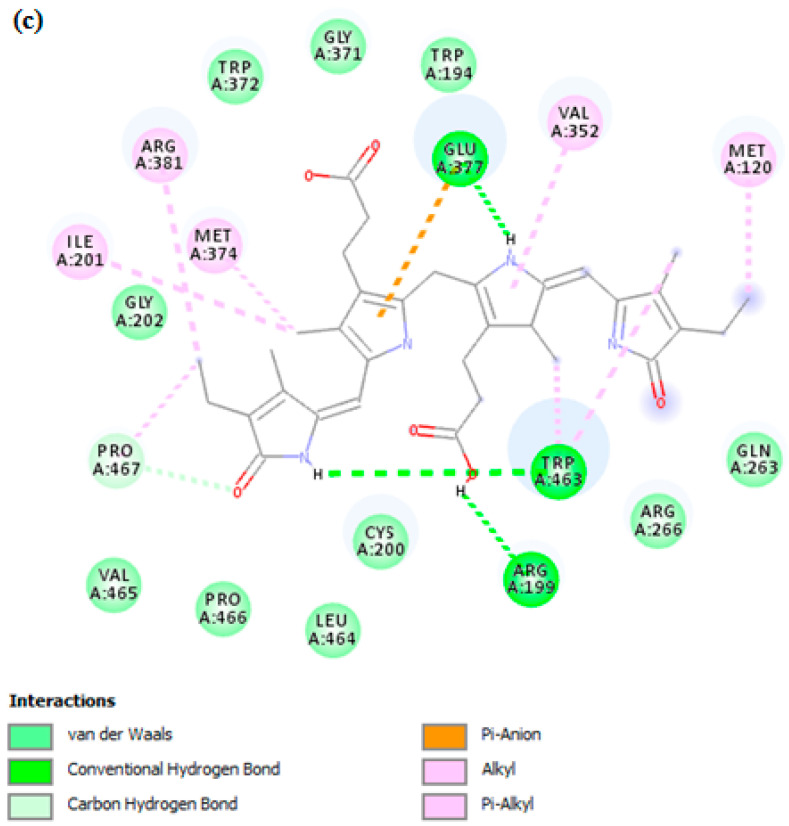
The 3D (**a**,**b**) and 2D (**c**) views of the molecular interactions of PCB with the surrounding amino acids of NADPH oxidase (PDB: 2CDU) (**A**), SOD (PDB: 4MCM) (**B**), COX-2 (PDB: 3LN1) (**C**), and iNOS (PDB: 1NSI) (**D**).

We employed molecular docking to explore the potential binding interactions of PCB with the receptor binding site of the target proteins and to elucidate their associated inhibitory and protective actions. This computational technique is highly useful in structure-based drug design for its ability to predict and analyze how small molecules interact with biological targets. Table 2 shows the results for the intermolecular interactions of PCB with NADPH oxidase (PDB ID: 2CDU), SOD (PDB ID: 4MCM), COX-2 (PDB ID: 3LN1), and iNOS (PDB ID: 1NSI), and Figure 6 shows the 3D (a,b) and 2D © views of the molecular interactions of PCB with NADPH oxidase (A), SOD (B), COX-2 (C), and iNOS (D). PCB demonstrated appropriate interactions with multiple acid residues for NADPH oxidase and SOD in the 2D and 3D views, as illustrated in Figure 6A,B. Our molecule fits precisely with NADPH oxidase and SOD, as confirmed by the binding energies of −8 kcal/mol and −6.4 kcal/mol, respectively (Table 2).

In fact, intermolecular conventional hydrogen bonding is recognized as the most crucial factor in demonstrating the stability of the docking complex and, consequently, the ligand’s inhibitory effect on the protein. PCB shares several intermolecular interactions with NADPH oxidase, such as five conventional hydrogen bonds with the oxygen (OH group) ASN350, THR291, SER293, SER326, and TYR296, and one H-bond with NH of TYR296 (Figure 6A). Additionally, 2D views show attractive van der Waals forces colored light green with ASN294, ASP260, TYR288, SER328, TYR159, and LEU299, and the pi-sigma binding type with the LEU346 and ALA295 amino acids (Figure 6A). In addition, eight are alkyl and pi-alkyl bonds with the amino acid residues LYS187, PRO298, TYR188, LEU259, and ILE297 (Figure 6A).

In addition, for SOD we observed four hydrogen bonds with GLN22, SER102, SER105, and SER25 (Figure 6B). The 2D views also demonstrated attractive van der Waals forces with VAL29, GLY27, ARG69, GLU77, ASP109, GLY108, ASN26, SER107, PRO28, ASP101, and ILE104 (Figure 6B). Additionally, there are three interactions involving alkyl and pi-alkyl bonds with the amino acid residues LEU67, HIS110, and VAL103 (Figure 6B).

Regarding COX-2, Figure 6C shows various interactions of PCB with the target protein. The corresponding binding energy is −8.3 kcal/mol. Two hydrogen bonds are formed between the OH group of the ligand, such as SER457 and ARG455, with another H-bonding with ASN28 (NH group). Additionally, we observed several van der Waals bonds with the GLY48, THR61 and 47, ARG46, LYS459, LEU109 and 458, LYS454, GLN529, and PHE456 amino acid residues (Figure 6C). We also detected Pi Pi T-shaped, alkyl, and Pi-alkyl interactions with ARG29, TYR108, LYS68, LEU65, and PHE49 (Figure 6C). These interactions are involved in complex stabilization and contribute to its inhibitory effect.

Finally, we studied the interactions of PCB with iNOS, encoded as 1NSI.pdb (Figure 6D). The binding energy was −9.1 kcal/mol. Three conventional hydrogen bonds were observed with GLU377, TRP463, and ARG199. Several van der Waals interactions were noted with the VAL465, PRO466, LEU464, CYS200, GLY202, GLY371, TRP194, TRP372, ARG266, and GLN263 amino acids (Figure 6D). Additionally, one carbon-hydrogen bond was detected between PCB and PRO467, and one pi-anion bond was observed with GLU377 (Figure 6D). Eight alkyl and pi-alkyl bonds were formed between PCB and ILE201, ARG381, MET374, PRO467, VAL352, MET128, and TRP463 amino acid residues of the targeted protein in chain A (Figure 6D).

## 4. Discussion

The consumption of a Western diet can have adverse health consequences due, among other factors, to its high fat content. The latter causes lipotoxicity, which promotes oxidative stress, inflammation, and tumorigenesis [54], also altering the integrity of the intestinal epithelial barrier. These alterations to the intestinal barrier can also allow bacterial LPS to enter systemic circulation, triggering immune responses, chronic inflammation, and contributing to the development of metabolic diseases [55]. In this study, we wanted to see whether the microalga spirulina, which has been demonstrated to have anti-inflammatory and antioxidant actions on several disease models, was able to ameliorate LPS- and/or lipid-induced cytotoxicity and intracellular deleterious processes in intestinal epithelial cells, thus eventually protecting the intestinal barrier from these insults. For this purpose, we used a well-established cellular model of enterocytes, the human Caco-2 intestinal cells, on which we assessed the protective properties of SPAE against LPS- and/or LM-induced damage.

As expected, LPS (1 μg/mL) and/or LM decreased cell viability and induced cell hyperpermeability compared with the control group. These observations corroborate several previous investigations in which treatments with LM and LPS resulted in inflammation and increased cell permeability [56]. SPAE showed no cytotoxic effects. On the contrary, SPAE was cytoprotective, improving Caco-2 cell viability after LPS, LM, and LPS + LM. Moreover, SPAE also protected against membrane hyperpermeability induced by LPS and/or LM. These findings agree with previous ones in other cellular models [57,58]. Furthermore, several investigations have indicated that LPS triggers an inflammatory signaling cascade to reduce tight junction protein expression, leading to increased intestinal permeability and disruption of intestinal epithelial barrier function [59]. We detected decreased expression of tight junction protein genes (ZO-1 and OCL-1) in post-confluent Caco-2 cells after LPS and/or LM, and these changes were partially reversed by SPAE.

The endoplasmic reticulum is responsible for the synthesis, folding, and processing of secretory and transmembrane proteins [60]. A high-fat diet induces intestinal hyperpermeability and oxidative stress in the endoplasmic reticulum of intestinal caliciform cells, as well as activation of the inflammatory signal [61]. Indeed, the mucin glycoprotein (Muc2) is produced by intestinal caliciform cells and is susceptible to misfolding, stimulating the developing protein response with the aim of restoring homeostasis. Failure to restore misfolding induces stress and triggers an inflammatory response and apoptosis, reducing barrier integrity [62,63]. Activation of iNOS during intestinal inflammation has also been clearly demonstrated in previous studies [64]. Of note, we found that incubation of Caco-2 cells with LPS and/or LM induced overexpression of genes related to ER stress (XPB-1 and CHOP) as well as iNOS. Interestingly, SPAE down-regulated iNOS expression and decreased ER stress markers, suggesting an ability of SPAE to counteract these deleterious processes.

LPS, LM, and their combination induced a state of oxidative stress in cultured Caco-2 cells, as proven by a significant increase in MDA levels, a significant depletion in the activity of antioxidant enzymes such as SOD, CAT, and GPx, as well as a decrease in the levels of non-enzymatic antioxidants such as thiol groups and reduced glutathione. Spirulina possesses an antioxidant defense system capable of eliminating reactive oxygen species that can damage cells by causing oxidative stress [65]. This antioxidant system reduces most oxidized forms, and the antioxidant activity of spirulina extract is associated with certain phycobiliproteins, such as C-phycocyanin and allophycocyanin [66]. Of note, in our study spirulina showed remarkable activity in significantly preventing cell membrane lipoperoxidation and in restoring the antioxidant balance after both LPS and LM, underscoring its crucial role in maintaining oxidative homeostasis and neutralizing ROS by significantly reducing their overproduction at the cellular level. Similar pharmacological analyses have demonstrated spirulina’s beneficial properties in animal models of obesity and fatty liver [27,67].

A high-fat diet leads to increased expression of inflammatory cytokines such as TNF-α and IL-1β in visceral adipose tissue, facilitating LPS entry [10]. It has also been shown that exposure to TNF-α increases the para-cellular permeability of a monolayer of differentiated Caco-2 cells [68]. Interestingly, Chen et al. (2012) [57] showed that c-phycocyanin results in inhibition of NO release, activation of NF-κB, and suppression of TNF-α formation. In another previous study, spirulina was shown to prevent LPS-induced up-regulation of iNOS, COX-2, TNF-α, and IL-6 in BV-2 cells [69]. We have detected a significant increase in pro-inflammatory cytokines in the basal medium of the Caco-2 cell monolayer, notably IL-1β, IL-6, and TNFα, compared with the control group. These effects were more pronounced in cells treated with the LPS + LM combination than in the LPS and LM groups. Importantly, SPAE protected against LPS- and/or LM-induced increases in IL-1β, IL-6, and TNFα. These findings underscore spirulina’s potent anti-inflammatory activity [70], highlighting its potential as a protective agent under conditions where inflammation is exacerbated by the combination of several factors such as fatty acids and LPS [71,72].

Regarding the in vivo relevance of our findings, we acknowledge that translating in vitro concentrations to in vivo doses can be challenging due to differences in absorption, metabolism, and distribution. While further in vivo studies are necessary to confirm effective dosages, our findings from Arrari et al. [29] suggest that SPAE at a dose of 500 mg/kg effectively elicits measurable biological responses, such as reducing oxidative stress and providing protective effects against obesity and colitis in Wistar rats. Taken together, all these results support the idea of spirulina’s remarkable efficacy as an adjuvant for the treatment of medical conditions that involve inflammation and oxidative stress, like obesity [73].

To further support our in vitro results, we conducted molecular docking studies to explore the binding of the main active compound of spirulina, PCB, to selected antioxidant and anti-inflammatory enzymes. PCB demonstrated strong antioxidant potential, comparable to other phytochemicals [74]. As one of Spirulina’s most significant bioactive compounds, PCB is notable for its potent antioxidant and anti-inflammatory properties [75]. An in vitro study identified PCB as the primary compound in spirulina responsible for its antioxidant effects [51]. A biliverdin derivative in spirulina mimics bilirubin in inhibiting NADPH oxidase activity [76]. This inhibition is significant because NADPH oxidase is a primary source of ROS involved in oxidative stress, particularly in mammalian cells [77], and contributes to PC12 cell survival [78]. In experimental settings, PCB reduced oxidative damage and supported nitric oxide bioactivity, helping to preserve and enhance SOD functionality [78,79], which detoxifies superoxide radicals. This overall reduction in oxidative stress can help mitigate oxidative damage to cells and tissues [25,80].

Regarding antioxidants, we selected NADPH oxidase (PDB ID: 2CDU) due to its critical role in generating ROS [81] and superoxide dismutase (SOD) (PDB ID: 4MCM), a key antioxidant enzyme that protects cells against ROS toxicity [29]. Interestingly, we observed a strong binding affinity of PCB with NADPH oxidase, which suggests it is specific and biologically relevant. NADPH oxidase is a significant source of ROS, as it transfers electrons from NADPH to molecular oxygen to produce superoxide radicals. PCB could inhibit NADPH oxidase by blocking its activation or interfering with its subunits’ assembly, thereby decreasing ROS generation. This inhibition may help prevent oxidative stress, which is a key factor in inflammation and tissue damage [75]. PCB binding to SOD was moderate but theoretically strong enough to be biologically significant. We did not detect an inhibitory effect of SPAE in SOD activity but an ability to counteract LM- and/or LPS-induced damage. PCB could enhance the activity of SOD by directly binding to it and stabilizing its structure, thereby enhancing its ability to catalyze the dismutation of superoxide radicals (O^2−^) [82]. This would reduce the buildup of reactive oxygen species (ROS), which can be harmful in excess. By increasing SOD activity, PCB could contribute to maintaining oxidative balance within cells and tissues [83]. Several in silico studies on NADPH oxidase and SOD have reported binding energies ranging from −6.27 kcal/mol to −10.62 kcal/mol [84,85] and from −4.5 kcal/mol to −9.18 kcal/mol, respectively [86,87]. These findings are consistent with our in silico observations. Moreover, our results show that PCB shares a relevant number of intermolecular interactions with NADPH oxidase and SOD, which support the stability of the docking complex and, consequently, the ability of PCB to induce a biologically relevant action on NADPH oxidase and SOD. These results further support the action of PCB in SPAE as an effective antioxidant in Caco-2 cells. Interestingly, dextromethorphan, a reference drug, exhibited a similar binding energy (−8.0 kcal/mol) with NADPH oxidase, while L-ascorbate, a potent standard antioxidant drug, showed a lower binding energy with SOD compared to our ligand (−5.3 kcal/mol) [87]. Dextromethorphan also demonstrated intermolecular interactions with Tyr188, Pro298, and Leu299 in NADPH oxidase via hydrophobic pi-alkyl bonds [88] and with Asn26, Ser109, Gly108, Cys117, Asn65, Val81, Val103, Ser102, Val29, Gln22, Phe20, His110, Ile112, Ile114, and Asp101 in SOD [87].

Regarding anti-inflammatory activity, we selected COX-2, which plays a key role in catalyzing the formation of prostaglandins [89], and iNOS, which is responsible for immune defense against pathogens and produces nitric oxide from L-arginine [90]. Our in silico findings demonstrated that PCB binds with high affinity to iNOS and COX-2, also sharing intermolecular interactions potentially stabilizing the docking complex. The anti-inflammatory properties of PCB could involve its ability to reduce the expression of COX-2 and iNOS or directly inhibit their enzymatic activities. By down-regulating COX-2 activity, PCB can reduce the production of pro-inflammatory molecules, alleviating inflammatory responses [91]. These findings are consistent with other studies and support the anti-inflammatory effects of PCB from spirulina [26,92]. Additionally, our results demonstrate the effectiveness of our compound compared to other docked molecules, such as mygalin [93], and compounds from *Olea europaea* L. leaf extract [94]. Notably, it performed better than the reference drug Celecoxib, which had a binding energy of −6.6 kcal/mol and formed three hydrogen bonds with Arg106, Gln178, and Leu338 [94].

Altogether, the docking analysis results confirmed that the tested PCB compound showed a strong binding affinity for NADPH oxidase, SOD, COX-2, and iNOS enzymes, with promising binding patterns comparable to those of L-ascorbate, Dextromethorphan, and Celecoxib, thus supporting the notion that PCB could be mediating the antioxidant and anti-inflammatory effects of SPAE on Caco-2 cells.

This study has several limitations. On one hand, Caco-2 cells are a human colonic carcinoma cell line and, although commonly used to study intestinal cell physiology, may not fully represent normal intestinal physiology. Verification in alternative models, such as intestinal organoids derived from healthy human tissue or additional cell lines like HT29, would strengthen the findings and improve the generalizability of these results. Secondly, although our in silico study was limited to PCB, the main and more studied antioxidant and anti-inflammatory compound found in spirulina, we cannot rule out the possibility that other minor components such as vitamins, polysaccharides, or polyphenols in SPAE are also exerting protective actions on Caco-2 cells. Finally, the effects of SPAE on tight junction (ZO-1 and OCL-1) and ER stress (XPB-1 and CHOP) markers were assessed only by real-time qPCR and not by Western blot.

## 5. Conclusions

Our results indicate that SPAE has no cytotoxic effect on cultured Caco-2 cells, suggesting its safety at concentrations of up to 500 µg/mL. Furthermore, SPAE significantly impacted epithelial barrier permeability by reducing LPS and/or LM-induced paracellular hyperpermeability. This hyperpermeability was associated with oxidative stress, evidenced by significantly increased levels of MDA and ROS, as well as a depletion in the antioxidant activities of SOD, CAT, GPx, GSH, and thiol groups. All these markers of oxidative stress were attenuated after SPAE treatment. At the molecular level, SPAE also restored the transcriptional overexpression of ER stress markers (XBP-1 and CHOP), the expression of tight junction proteins (ZO-1 and OCL-1), and iNOS expression induced by LPS and/or LM. Additionally, SPAE modulated the production of pro-inflammatory cytokines such as IL-1β, IL-6, and TNFα, demonstrating its efficacy in suppressing inflammatory responses. Our in silico analysis revealed molecular docking interactions of PCB with antioxidant and anti-inflammatory enzymes, showing effective negative binding energies comparable to those of reference antioxidants and anti-inflammatory drugs.

## Figures and Tables

**Table 1:** List of primers.

Gene	Sequence (5′-3′)
XBP1Fow XBP1 Rev	TGGCCGGGTCTGCTGAGTCCG ATCCATGGGGAGATGTTCTGG
CHOP Fow CHOP Rev	AGAACCAGGAAACGGAAACAGA TCTCCTTCATGCGCTGCTTT
ZO-1 Fow ZO-1 Rev	GACCTTGAGCAGCCGTCATA CCGTAGGCGATGGTCATAGTT
OCL-1 Fow OCL-1 Rev	CTTTGGCTACGGAGGTGGCTAT CTTTGGCTGCTCTTGGGTCTG
iNOSFow iNOSRev	TGCAGACACGTGCGTACTC GGTAGCCAGCATAGCGGATG
GAPDH Fow GAPDH Rev	CATGGCCTTCCGTGTTCCTA CCTGCTTCACCACCTTCTTGAT

**Table 2:** Docking energy, number of conventional hydrogen bonds, and binding-site residues of NADPH oxidase, COX-2, iNOS, and SOD with PCB using Autodock vina.

		Intermolecular Interactions	
Protein	Docking Energy (kcal/mol)	Conventional Hydrogen Bonds	Interacting Amino Acid Residues
NADPH oxidase	−8	6	SER293, THR291, ASN350, TYR288, LEU346, SER326, ILE297, LYS187, PRO298, TYR188, TYR296, LEU259, ALA295
COX-2	−8.3	3	LYS68, LEU65, PHE49, ASN28, SER454, ARG455, TYR108, ARG28
iNOS	−9.1	3	GLU377, MET374, ARG381, ILE201, PRO465, ARG199, TRP463, VAL352, MET120
SOD	−6.4	4	VAL29, GLY27, GLN22, LEU67, HIS110, VAL103, ARG69, GLU77, ASP109, GLY108, SER105, ASN26, SER107, PRO28, SER105, ASP101, ILE104, SER102

## Data Availability

The data presented in this study are available on request from the corresponding authors due to legal reasons. The dataset is part of an ongoing project and not yet finalized for public release.

## Abbreviations

CAT (Catalase); COX-2 (Cyclooxygenase 2); ER (Endoplasmic reticulum); FD4 (FITC-labeled dextran 4); GPx (Glutathione peroxidase); GSH (Reduced glutathione); H_2_O_2_ (Hydrogen peroxide); iNOS (Inducible nitric oxide synthase); LM (Lipid micelles); LPS (Lipopolysaccharides); LSD (Least significant difference); MDA (Malondialdehyde); NADPH (Nicotinamide adenine dinucleotide phosphate); OCL-1 (Occludin); PCB (Phycocyanobilin); PDB ID (Protein data bank identification code); ROS (Reactive oxygen species); SOD (Superoxide dismutase); SPAE (Spirulina Aqueous Extract); ZO-1 (Zonula occludens-1).
